# Autofluorescence detection and co-axial projection for intraoperative localization of parathyroid gland

**DOI:** 10.1186/s12938-022-01004-8

**Published:** 2022-06-16

**Authors:** Wei Chen, Xiaopeng Ma, Pengfei Shao, Peng Liu, Ronald X. Xu

**Affiliations:** 1 Department of Precision Machinery and Precision Instrumentation, University of Science and Technology of China, Hefei, 230026 China; 2 First Affiliated Hospital, University of Science and Technology of China, Hefei, 230031 China; 3 Suzhou Institute for Advanced Research, University of Science and Technology of China, Suzhou, 215123 China

**Keywords:** NIR, Parathyroid gland, Autofluorescence, Coaxial projection

## Abstract

**Background:**

Near-infrared (NIR) autofluorescence detection is an effective method for identifying parathyroid glands (PGs) in thyroidectomy or parathyroidectomy. Fiber optical probes provide quantitative autofluorescence measurements for PG detection owing to its high sensitivity and high excitation light cut-off efficiency at a fixed detection distance. However, an optical fiber probe lacks the imaging capability and cannot map the autofluorescence distribution on top of normal tissue background. Therefore, there is a need for intraoperative mapping of PGs with high sensitivity and imaging resolution.

**Methods:**

We have developed a fluorescence scanning and projection (FSP) system that combines a scanning probe and a co-axial projector for intraoperative localization and in situ display of PGs. Some of the key performance characteristics, including spatial resolution and sensitivity for detection, spatial resolution for imaging, dynamic time latency, and PG localization capability, are characterized and verified by benchtop experiments. Clinical utility of the system is simulated by a fluorescence-guided PG localization surgery on a tissue-simulating phantom and validated in an ex vivo experiment.

**Results:**

The system is able to detect indocyanine green (ICG) solution of 5 pM at a high signal-to-noise ratio (SNR). Additionally, it has a maximal projection error of 0.92 mm, an averaged projection error of 0.5 ± 0.23 mm, and an imaging resolution of 748 μm at a working distance ranging from 35 to 55 cm. The dynamic testing yields a short latency of 153 ± 54 ms, allowing for intraoperative scanning on target tissue during a surgical intervention. The simulated fluorescence-guided PG localization surgery has validated the system’s capability to locate PG phantom with operating room ambient light interference. The simulation experiment on the PG phantom yields a position detection bias of 0.36 ± 0.17 mm, and an area intersection over unit (IoU) of 76.6% ± 6.4%. Fluorescence intensity attenuates exponentially with the thickness of covered tissue over the PG phantom, indicating the need to remove surrounding tissue in order to reveal the weak autofluorescence signal from PGs. The ex vivo experiment demonstrates the technical feasibility of the FSP system for intraoperative PG localization with accuracy.

**Conclusion:**

We have developed a novel probe-based imaging and navigation system with high sensitivity for fluorescence detection, capability for fluorescence image reconstruction, multimodal image fusion and in situ PG display function. Our studies have demonstrated its clinical potential for intraoperative localization and in situ display of PGs in thyroidectomy or parathyroidectomy.

## Background

Thyroid nodules are among the most common diseases in adults, with a 20.43% prevalence by population, according to a survey of 78,470 participants across 31 provincial regions in China [1]. Up to 7%-15% of thyroid nodules are malignant tumors [2], and this number is increasing every year. Surgical procedures, such as total and partial thyroidectomies, remain the best treatment options for malignant thyroid tumors. In thyroid surgery, it is crucial to localize PGs intraoperatively to minimize incidental injuries. The PGs are two pairs of oval-shaped glands locate behind the left and right sides of the thyroid. Owing to their small size (ranging from 3 to 8 mm), it is challenging to locate them properly in a thyroid surgery [3]. In several studies, incidental parathyroidectomy rates range from 16.4% to 23% [4–7]. Patients may experience symptoms such as tingling fingers and feet, cramping in the hands after thyroid surgery due to transient hypocalcemia, which may occur in as many as 34% of cases [8, 9].

Up to date, a wide range of methods and approaches have been developed for accurate PG detection and location, nevertheless, none of them provide a satisfactory solution for intraoperative applications. The commonly used diagnostic tools, such as neck ultrasound and Tc99 sestamibi scintigraphy, have high sensitivity to detect PG adenomas. However, they can only be used preoperatively and have a low detection rate for normal small PGs [10, 11]. Additionally, Tc99 is radioactive. The immune colloidal gold technique is another method for PG confirmation based on the difference in parathyroid hormone levels between PGs and other tissues. These tissues can be distinguished intraoperatively in 6 min, however, a high false positive rate of 20% is possible [12, 13]. Frozen sections and pathology have high accuracy and are the gold standard for clinical PG confirmation, but it will take 20 to 30 min to get the final results [14, 15]. Some exogenous indicators such as carbon nanotube, methylene blue and ICG are found the potential for intraoperative PG confirmation. However, none of this agent is highly specific, and there are risks of allergy and tissue color staining [16–18]. Besides, immune colloidal gold technique, frozen sections and exogenous indicators are all invasive assays. In summary, without an effective method for intraoperative identification of PGs, the current clinical practice still largely depends on the subjective experience of surgeons [19].

In 2011, Paras et al. discovered autofluorescence emission of PGs with an emission peak at around 825 nm under the illumination of NIR laser and found that fluorescence intensity of PGs is more than 1.2 times higher than that of surrounding tissues [20]. Based on this mechanism, various devices have been developed and clinically tested for intraoperative identification of PGs. These devices can be categorized into fiber probe-based systems (e.g., PTeye, Medtronic, US) and imaging-based systems (e.g., PDE-Neo II, Hamamatsu, Japan). In a clinical study that compares the performance of an imaging-based system and a probe-based system, the probe-based system shows higher sensitivity and accuracy compared with the imaging-based system, which is critical when assessing the extremely weak autofluorescence signals from PGs [21]. Additionally, some of other advantages of a probe-based systems include immunity to interference of the ambient light, the excitation light reflection by surgical devices as well as other fluorescence sources such as surgical drapes, and invulnerability to the spatial variation of light illumination by point-by-point measurement [22, 23]. Despite all these advantages, probe-based system still lacks the intuitive imaging display of fluorescence intensity distribution. In the surgical scenario when the suspicious region of autofluorescence needs to be compared with other regions repetitively for confirmation, a conventional probe cannot “memorize” the location and the fluorescence intensity of each detected lesion. In comparison, a system that facilitates orthotopic display of fluorescence intensity even after the probe is moved away from the suspicious region will provide both fluorescence intensity distribution and high detection sensitivity, so that the surgeon is able to compare among multiple regions in order to localize the PGs and make appropriate surgical decisions.

In situ projection of autofluorescence signal offers a potential solution for intraoperative imaging guidance by a probe-based system. The concept of in situ projection of fluorescence emission is first proposed by Sarder et al. [24]. McWade et al. employs the projection technique for PG localization and build an overlay tissue imaging system (OTIS) for enhanced visualization of PGs so that surgeons do not need to frequently switch their sight of view between the surgical field of view (FOV) and the monitor [25]. In contrast to a conventional probe-based system, the OTIS system has the advantage of non-contact measurement as an imaging-based system. Thus, it can be used without sterilization when the working distance is usually set at 35 cm to 50 cm to keep the non-sterile cameras outside the recommended sterile zone in surgery. However, the OTIS system places a NIR camera and a projector side by side with a mismatched surgical view, making it necessary to calibrate the system at different working distances [26, 27]. We have previously developed a co-axial projective imaging (CPI) module that accurately matches the camera scene and the projector scene automatically at any working distances, which forgo the necessity of the complicated calibration process [28]. In this paper, we integrate CPI with PG autofluorescence detection probe and propose a novel FSP navigation system for intraoperative mapping and display of PGs with high sensitivity and high spatial resolution for imaging. The engineering parameters and performance characteristics of our system are characterized by benchtop experiments. It is demonstrated that the novel system has the clinical potential for intraoperative localization of PGs in thyroidectomy or parathyroidectomy.

## Results

### FSP system performance characterization

**Fig. 1:**
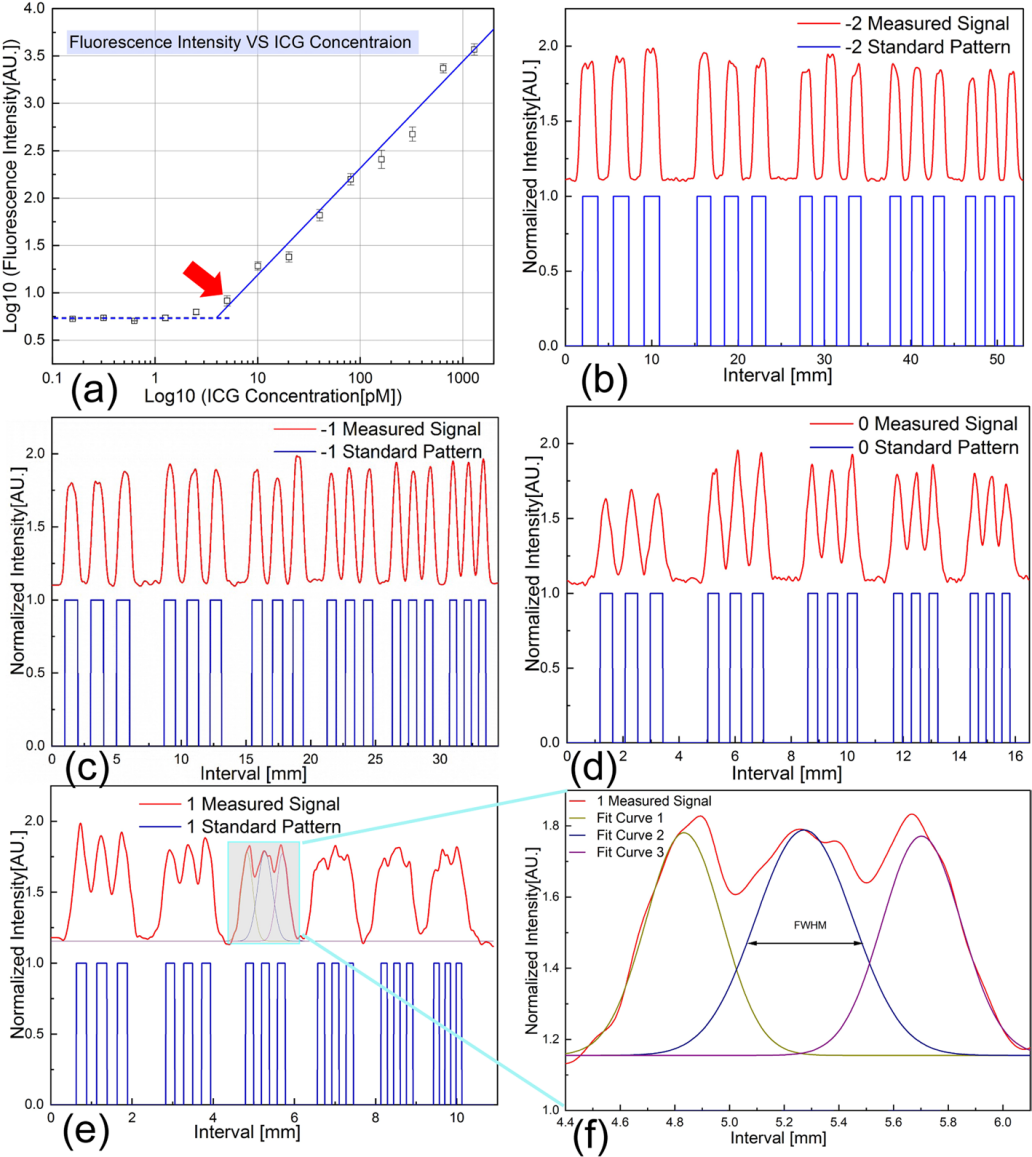
Fluorescence detection sensitivity and spatial resolution for detection characterized by ICG solution and resolution target. **a** Sensitivity is about 5 pM ICG solution, marked by red arrow. Excitation light power is about 20 mW focused at an area of a 200 μm diameter, with a power density of about 0.63 W/mm^2^. **b** Resolution test: Group -2 data. **c** Group -1 data. **d** Group 0 data. **e** Group 1 data. **f** FWHM calculation using Gaussian fit, spatial resolution defined by Sparrow criterion. Element 3 of group 1 is discernible, which indicates spatial resolution for detection is about 397 μm

Since PGs exhibit a weak autofluorescence intensity, it is important to design a system of high SNR in order to remove the interference of excitation light source and reveal the fluorescence emission. Considering that ICG shows the NIR fluorescence emission peak similar to that of PGs, it is commonly used for quantitative verification of PG detection [23]. According to Fig. 1a, the log of ICG concentration has a proximate linear relationship with the log of fluorescence intensity in FSP system. In addition, the system is able to detect an ICG concentration of as low as 5 pM [23], superior to many commercially available NIR imagers [29], indicating its technical capability for PG detection.

To evaluate the spatial resolution for fluorescence detection, the detection probe is translated over the surface of a standard resolution target involving four groups of elements: Group -2, Group -1, Group 0 and Group 1 (Fig. 1b–e). The signal peaks are fitted by Gaussian distribution and full width at half-maximum (FWHM) is calculated using commercial software OriginPro (OriginLab, US). According to Fig. 1f, the smallest discernible element is the third element in Group 1. According to the Sparrow criterion, the spatial resolution for fluorescence detection is about 397 μm (Dmin= 0.85*FWHM), indicating its clinical capability for distinguishing between different PGs.

**Fig. 2:**
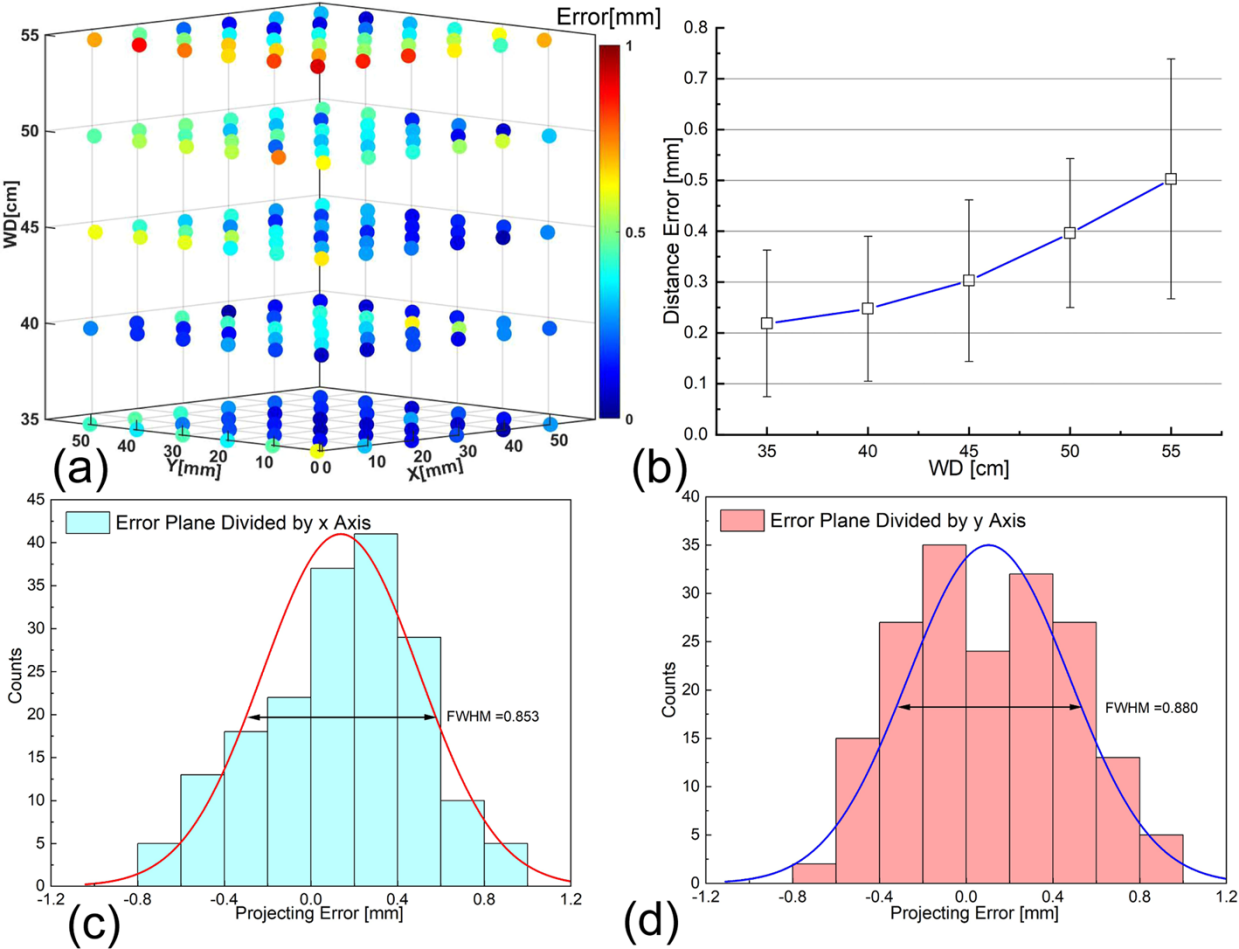
Projecting error at different working distance and spatial resolution for imaging measurement. **a** 3D distribution of projecting error at different working distance, the error is less than 0.92 mm. **b** Variation trend of distance error with working distance, maximal averaged error is 0.5 ± 0.23 mm. **c** and **d** Distance error distribution divided along the two orthogonal axes. Overall spatial resolution evaluation using Sparrow criterion, which indicates spatial resolution is 748 μm

The autofluorescence signal acquired by the FSP system is displayed in situ through the CPI module. Unlike a conventional projector, the CPI module has the advantage of high projection accuracy independent with its working distance [28], as demonstrated in Fig. 2 where the positioning and projection accuracies are evaluated at five different working distances. According to Fig. 2a, the FSP system has a projection bias of less than 0.92 mm at the working distance ranging from 35 to 55 cm. Despite the increasing of error with working distance (Fig. 2b), the maximal averaged error is 0.5 ± 0.23 mm, which still meets the requirements for intraoperative localization of PGs whose sizes range from 3 to 8 mm.

The overall spatial resolution for imaging is affected by the accuracy of probe positioning and in situ projection, which is reflected in the projection accuracy over a detection target. Projection errors along *x*-axis and *y*-axis are fitted by Gaussian distribution using commercial OriginPro software (OriginLab, US). According to Fig. 2c and Fig. 2d, the maximal FWHM along the two axes is 0.88 mm. Therefore, the spatial resolution for imaging based on the Sparrow criterion [30] is 748 μm when the working distance ranges from 35 to 55 cm. According to the above characterizations, FSP system has a high detection sensitivity and high spatial resolution for imaging, which exceeds requirements for PG visualization [23]. System’s dynamic performance measurement yields a time delay of 153 ± 54 ms for imaging, which indicates the surgeon could scan the target tissue in real-time.

### Surgical simulation on a PG-simulating phantom

**Fig. 3:**
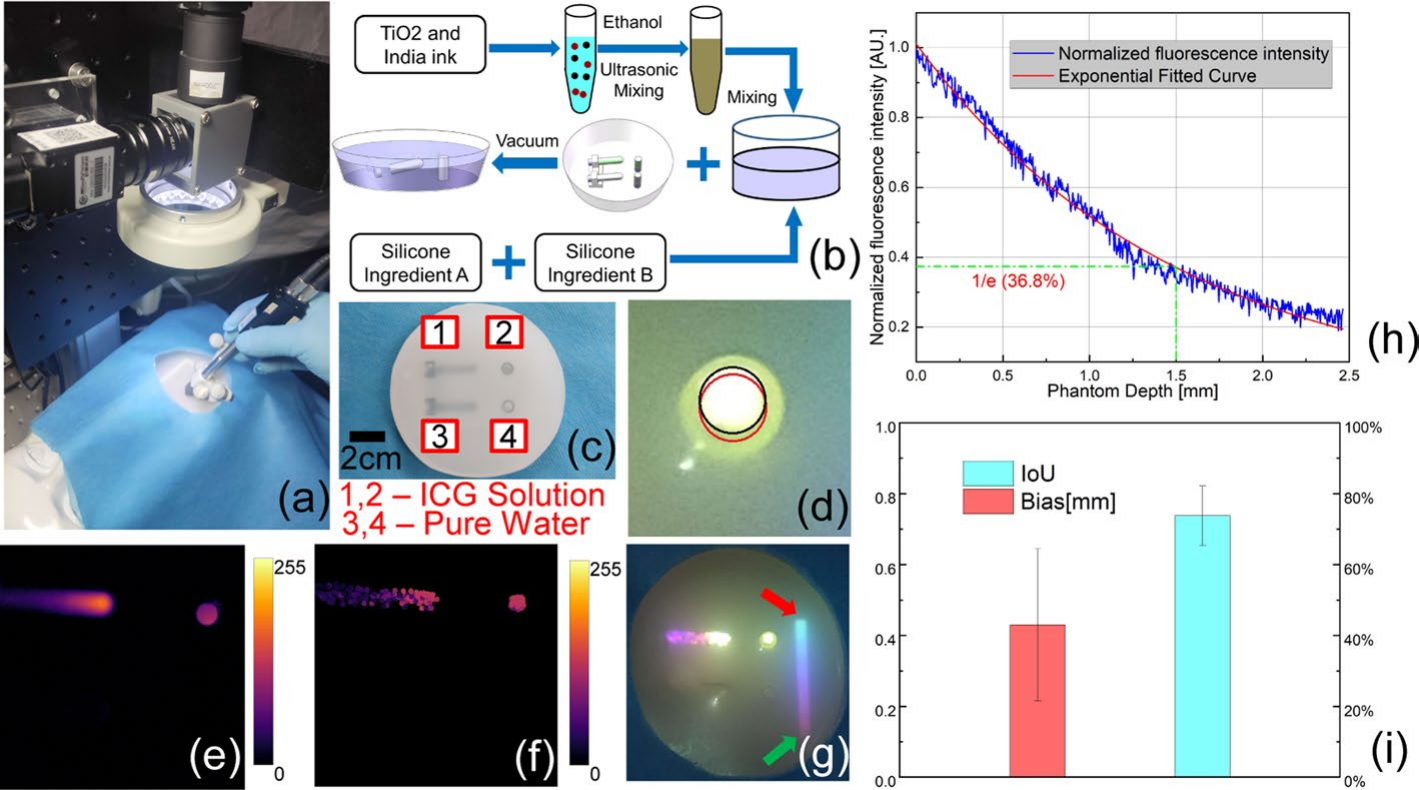
Surgical scenario simulation using human model and ICG solution phantom with operating room lights on. **a** An experienced user is detecting PG phantom simulated by ICG solution. **b** Phantom preparation process: ICG solution simulated PG and pure water as negative control buried in RTV silicone. **c** Phantom: cup 1 is installed at a tilt angle to simulate PG covered by adipose. Cup 2 is a flat-bottom cup to simulate PG exposed completely. Cup 3 and cup 4 are filled with water. **d** Simulated PG is delineated by FSP in situ projection image (area in black circle) and compared with the simulated PG area fitted based on the fluorescence image obtained by the LSFI. **e** Fluorescence intensity distribution measured by the LSFI. **f** Fluorescence intensity distribution measured by FSP. **g** Probability to be parathyroid gland represented by autofluorescence intensity normalized to background, in situ projected onto PG phantom by pseudo-color image, red arrow indicates higher probability and green arrow indicates lower probability. **h** Fluorescence intensity attenuates exponentially along cup 1 as thickness of overlapped silicone increases. (i) The center distance bias of fitted area is about 0.36 mm and area overlap represented by IoU is about 76.6% (n = 10)

The FSP system offers both the advantages of high sensitivity and spatial resolution for imaging while not being interfered by ambient light in the operating room. Figure 3a shows that an experienced user is using the detection probe to scan on a human model installed with PG phantom which is prepared according to the process in Fig. 3b and shown in Fig. 3c. The phantom fluorescence performance is characterized by a commercial laser scanning fluorescence imaging (LSFI) system (Biomolecular imager, GE Healthcare) and the fluorescence image is shown in Fig. 3e. The area marked by black circle in Fig. 3d is indicated as PG by FSP and it is compared with the red circle fitted based on the fluorescence image characterized by LSFI. The fluorescence intensity of simulated PG covered by adipose attenuates exponentially as the embedding depth in room-temperature vulcanizing (RTV) silicone increases. The fluorescence intensity value is only 36.8% of the highest when the depth is 1.5 mm (Fig. 3h), indicating that adipose over PG should be removed before weak autofluorescence signal could be detected. Figure 3f shows an example of fluorescence image measured by FSP. The normalized fluorescence intensity along with a color bar are projected onto the simulated surgical site, representing the probability distribution to be a PG (Fig. 3g). The end of color bar marked by a red arrow indicates a high probability to be PGs while the other end of color bar marked by a green arrow indicates a low probability. The overlap of the two areas located by FSP and LSFI, respectively, is evaluated using IoU, which is defined as the overlapped section of two areas divided by the total area and is widely employed as a metric to evaluate the performance for object detection [31]. The center distance bias of the located areas is about 0.36 mm and area overlap represented by IoU is about 76.6% (sample number = 10) (Fig. 3i), indicating the FSP’s capability for accurate intraoperative PG localization.

### Ex vivo* validation of PG detection and *in situ* projection*

**Fig. 4:**
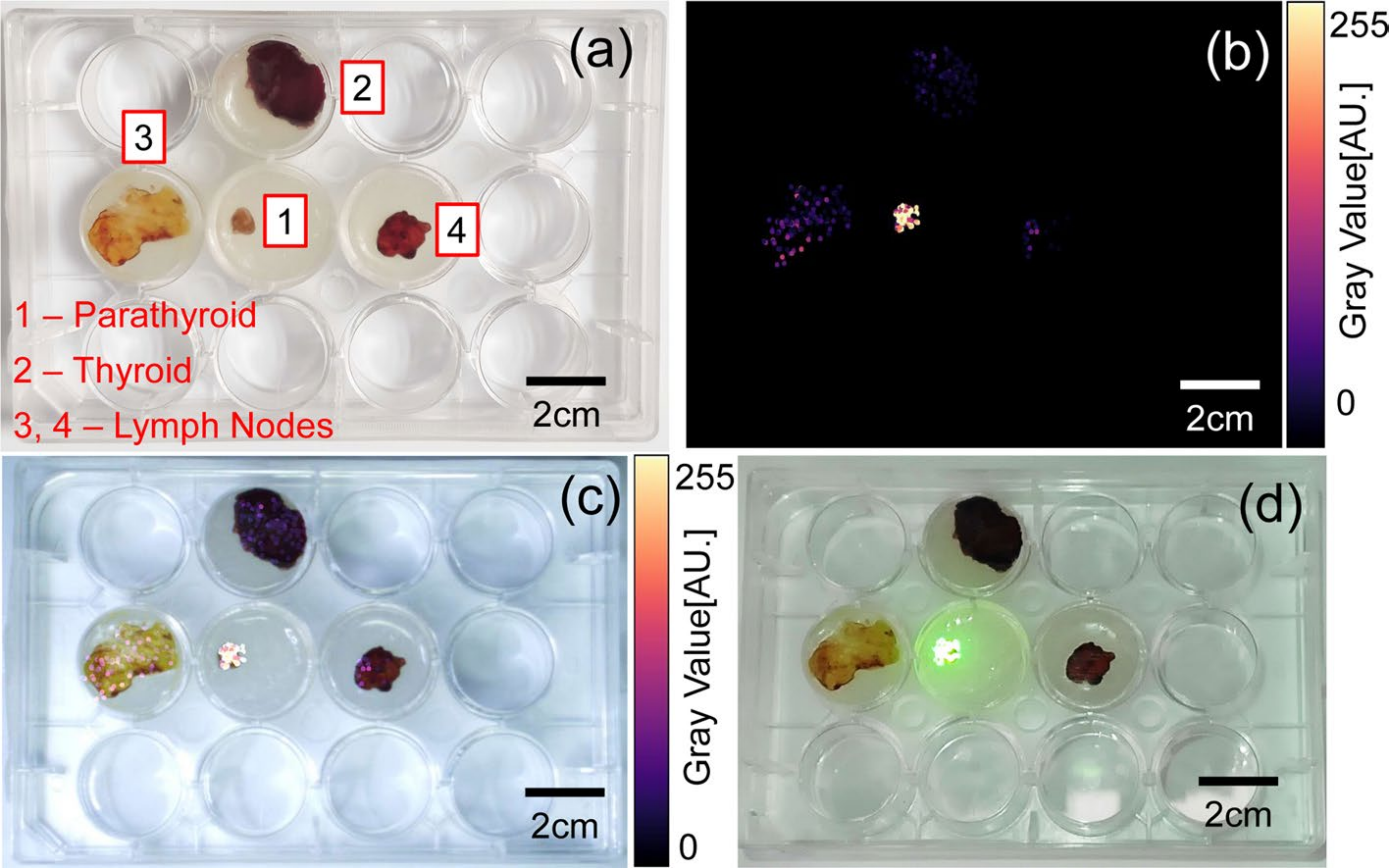
Ex vivo tissue validation. **a** Parathyroid, thyroid and lymph node tissues are fixed in a container. **b** Pseudo-color fluorescence intensity image displayed on a screen after the probe randomly scanned on each tissue. **c** Fusion image of pseudo-color fluorescence intensity and color FOV, which could be displayed on another monitor for surgical teleguidance. **d** Visible green light is projected only on PG and no green light overlaid on other kind of tissues

The FSP’s capability for PG detection and in situ display is validated in different tissue samples, including lymph nodes, thyroid glands, and PG, as shown in Fig. 4a. The cutoff for differentiating PG and other tissues is set to be 1.5 [23]. The process of detection is divided into two phases: (1) background measurement phase—as the probe is randomly scanned over certain positions of lymph nodes and thyroid glands, the maximal fluorescence intensity value is obtained as autofluorescence signal background; (2) detection phase—the real-time detected fluorescence intensity is normalized to the background, and the pseudo-color image of autofluorescence signal (Fig. 4b) is obtained based on the normalized fluorescence intensity distribution over the tissues. The image can be either fused with the RGB image of the background tissues for display on a local or remote monitor (Fig. 4c), or be binarized based on the cutoff and projected back onto the sample tissues (Fig. 4d). According to Fig. 4d, only the PG is overlaid with the green light, indicating the FSP’s technical feasibility for differentiating PGs from other tissues.

## Discussion

Autofluorescence detection was first reported in 2011 and has been proven to be a reliable method for intraoperative PG localization [20]. As two primary forms of intraoperative autofluorescence detection, imaging and fiber-based systems both have their own advantages and limitations. Generally speaking, an optical imaging system facilitates precise localization of the PGs during surgery, but has a relatively lower sensitivity [21]. On the other hand, a fiber optical system is able to achieve a higher sensitivity, but lacks intuitive mapping of the target PGs with respect to tissue background. We have previously developed a CPI module for in situ display of fluorescence intensity with a high projection accuracy of less than 1 mm in a working distance from 30 to 50 cm without the need for image registration [28]. In this paper, we propose a novel system to take the advantages of both optical imaging and fiber optics by integrating CPI with fiber optical detection and mechanical scanning.

In our proposed design of the FSP system, the CPI module comprised an RGB camera and a digital projector is to serve the following three important functions: (1) providing a true-color FOV for multimodal image fusion and teleguidance; (2) projecting the PG information and the surgical instruction orthotopically at the surgical site; (3) identifying the location and orientation of the FSP probe in real-time by tracking the fiducial markers on the probe. The fluorescence detection probe of the FSP device has also been deliberately designed in order to detect weak fluorescence signals with high SNR: (1) a filter with high cut-off optical density (OD), as an OD of 6 or more is chosen. (2) The stray light coming from the reflection of excitation light is collimated by a lens to obtain perpendicular incidence onto the filter surface, thus it is easier to remove the reflected light using suitable filters. Due to the elimination of interference from reflected excitation light, the probe could obtain higher SNR and fluorescence detection sensitivity compared with traditional imaging systems. (3) An avalanche photodiode (APD) is chosen as the fluorescence detector instead of traditional spectrometer or NIR camera due to its high quantum efficiency and better frequency response.

The performance characteristics of the FSP system are evaluated by detecting the key parameters for fluorescence mapping. Sensitivity for autofluorescence detection is measured using ICG solution of different concentration, a minimum 5 pM solution can be detected, which demonstrates not only the capability for PG localization, but it also exceeds the performance of many NIR imagers as reported [29]. Other factors that impact image reconstruction performance are also characterized, including spatial resolution for fluorescence detection, projection accuracy and spatial resolution for imaging. A fluorescence phantom made of a NIR laser diode (LD) and standard resolution target is used to characterize spatial resolution for detection. And it shows that the system has a minimum detection spatial resolution of about 397 μm, which meets the requirements for distinguishing between different PGs. The spatial resolution for imaging is about 748 μm which is better than the OTIS built by McWade which has already been proven for effective PG visualization intraoperatively. The projection accuracy is in 0–0.92 mm for our system and it is close to OTIS [25]. However, the projection accuracy characterization data for OTIS working beyond 35 cm are not presented, while FSP is characterized from 35 to 55 cm. In comparison with the FSP system, the OTIS system has the advantage of non-contact measurement, so it can be used directly without sterilization when the working distance is usually set at 35 cm to 50 cm to keep the non-sterile cameras outside the recommended sterile zone in surgery. If the working distance is less than 35 cm, it only needs to be wrapped in a transparent sterile wrapper. However, one of the disadvantage of the OTIS is that the excitation light and fluorescence intensity vary with the working distance [32], which will compromise the fluorescence imaging system’s performance: (1) the detected fluorescence signal cannot be quantified when it is acquired at different working distances; (2) a greater working distance as FSP has will require a higher power excitation light and a more sensitive image sensor. Higher power lasers might put the surgeons in dangerous situation while better performing sensors will cost more. In comparison, the fluorescence signal and detection sensitivity of FSP are not affected by the working distance. When a larger working distance is required, camera and projector lenses with longer focal length can be used to meet the requirements for larger field of view.

The simulation of fluorescence-guided PG localization surgery proves the FSP’ system’s capability for accurate PG delineation with operating room ambient lights on. The PG can be outlined by FSP scanning using the normalized fluorescence intensity which represents higher probability to be PGs when the intensity value is high. However, due to the strong absorption and scattering properties of biological tissues, autofluorescence attenuates exponentially with the thickness of covered adipose over PG phantom. Therefore, in order to detect PGs with weaker autofluorescence, the tissue over PGs should be removed before a successful detection, or we can design a probe so that surgeon can easily insert it into the tissue in future. Here, we have demonstrated the system’s capability for reconstruction of fluorescence image, features of multimodal image fusion with color FOV during the random scanning on target tissue. When a proper cutoff for PG judgement is set, PGs could be delineated and visible light will be in situ projected to differentiate them from other tissues.

Our work has demonstrated FSP to be a novel imaging device for autofluorescence detection. However, there are still some limitations for our work. Firstly, the FSP’s performance is only validated by ex vivo experiments in limited cases (one patient). Therefore, it is necessary to continue to validate the system's performance on more patients intraoperatively. Secondly, an accurate localization of PG for the high-sensitivity FSP system is based on the hypothesis that PG emits autofluorescence at least 1.5 times higher as the other tissues. However, previous work indicates that some colloid nodules and brown fat might also exhibit strong autofluorescence which could cause false positives [33, 34]. Therefore, there is still a lack of sensitivity and specificity data of in vivo PG detection for FSP system, which should be investigated in the future work.

## Conclusion

We build a novel autofluorescence imaging system which has the following features: high sensitivity for fluorescence detection, fluorescence image reconstruction, multimodal image fusion and in situ PG display function. Compared to previous probe-based and imaging-based detection systems, it is a novel imaging system for PG navigation intraoperatively. The systems’ performance is characterized: sensitivity for fluorescence detection is 5 pM ICG solution; spatial resolution for fluorescence detection is 397 μm; in situ display accuracy is 0.5 ± 0.23 mm and spatial resolution for imaging is 748 μm. Averaged dynamic time latency is 153 ± 54 ms, which proves the system’s capability for real-time detection. Fluorescence-guided PG localization surgery is simulated in the laboratory, validating the system’s capability to locate PG phantom with operating room ambient light interference. The simulation experiment yields a position detection bias of 0.36 ± 0.17 mm and IoU of 76.6% ± 6.4%, proving the system’s capability for PG location with accuracy. The system’s imaging capability is validated using ex vivo tissues and it has shown the performance for PG in situ display. Our benchtop validation has verified the novel imaging system’s feasibility, and it is demonstrated that the FSP system has great potential for clinical utility in future.

## Methods

### Design of FSP system

The intraoperative PG location FSP system is designed by integrating a point-by-point fluorescence scanning probe, an optical track module and an in situ display module. Autofluorescence intensity of the surrounding confirmed tissues such as lymph node and thyroid gland are measured as the background signal. Real-time detected fluorescence intensity during scanning is normalized to the background, and a map of fluorescence distribution is obtained when the detected signal is correlated with the surgical site. Since the probe is always in contact with the tissue during the real-time detection, the probe tip and the measured point is overlapped in position. The probe position is detected by the RGB camera in the CPI which monitors the surgical site and tracks the fiducial marker mounted on the probe. Since the relative position of the probe tip and the fiducial marker is known and fixed, once the pattern of marker is recognized and located in the image acquired in real time, the image coordinate of the probe tip in the camera image plane can be calculated, which also gives its image coordinate in the projection module. In this way, the fluorescence distribution map would be synthesized and displayed on a local or remote monitor in which situation teleguidances for remote surgeries are possible. The cut-off value is set to binarize the fluorescence distribution map and positive areas as PGs can be projected by visible light in situ over the tissue by the CPI module.

**Fig. 5:**
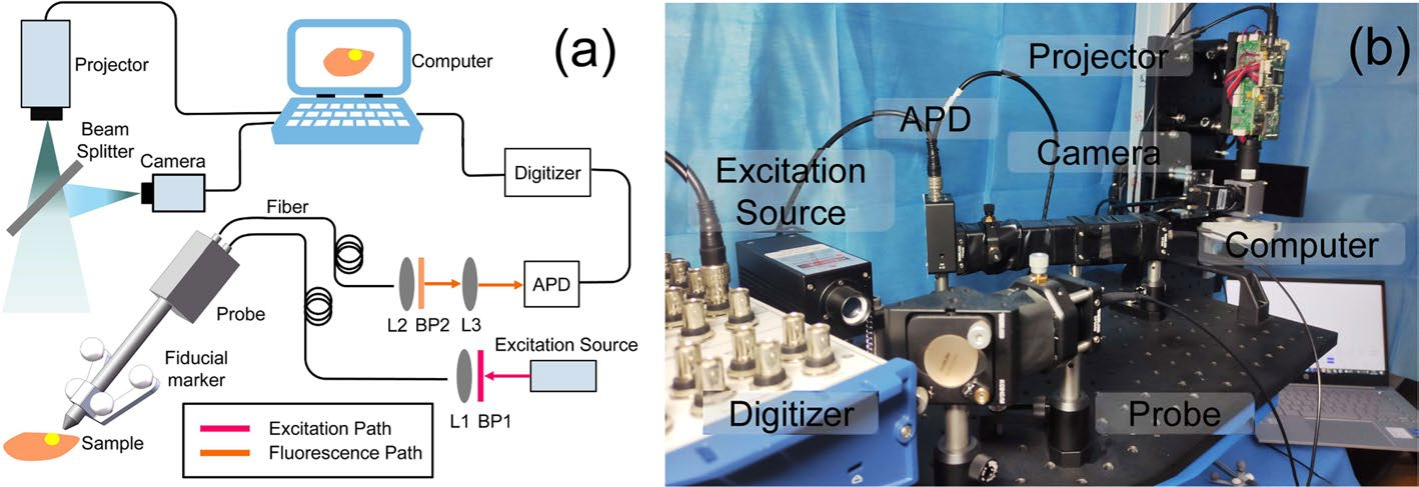
FSP system design. **a** Schematic of FSP system. **b** FSP system in laboratory environment

**Fig. 6:**
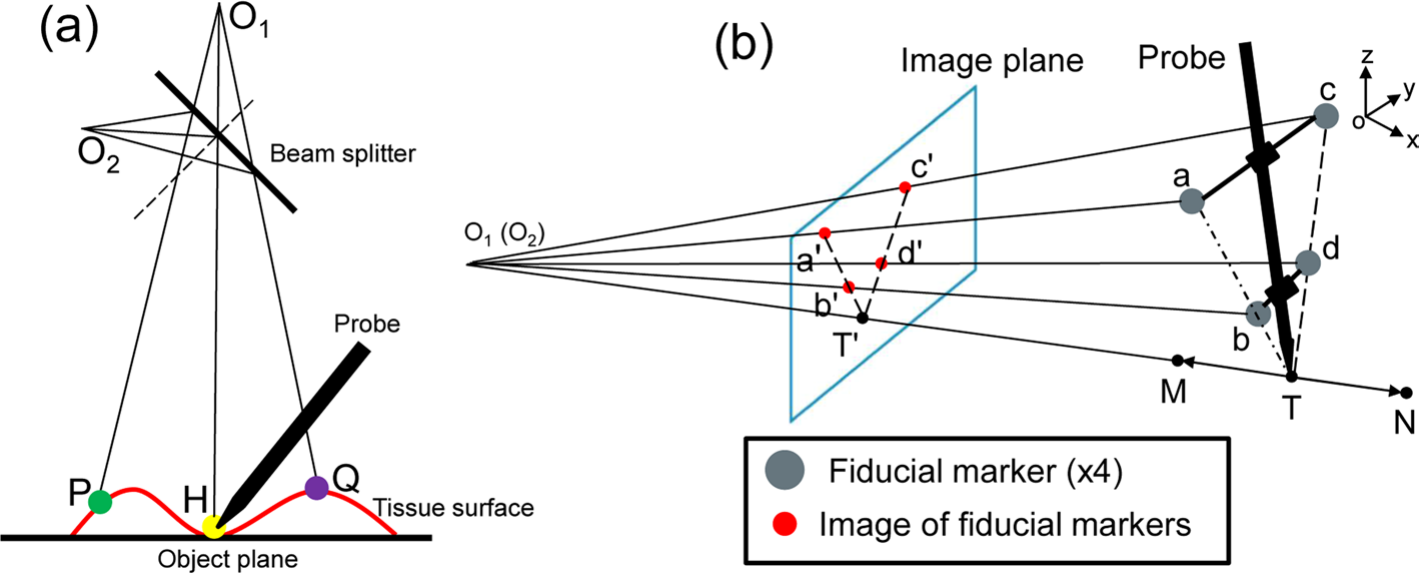
Illustration of 2D fluorescence projection to 3D cavities. **a** Optical paths of co-axial projector and camera system. **b** Probe tip detection by fiducial marker tracking, and point-by-point in situ projection

The hardware of the FSP system consists of two parts: a fluorescence detection probe and CPI module (Fig. 5a). The whole system is built on an optical table (Fig. 5b) which would also be a simulation of a surgical operating table. The probe comprises a 785-nm laser (Changchun New Industries, China), an excitation filter (Semrock, US), three aspherical lens (Thorlabs, US), two silica fibers (Nanjing Chunhui, China), a customized Raman probe (OCEANHOOD, China) installed with fiducial markers, a customized emission bandpass filter (Shenzhen Nanomacro, China), an APD (Thorlabs, US), a digitizer (National instruments, US) and a data processing laptop. 20-mW NIR 785-nm excitation laser is output from a Raman probe which focuses the light to be a spot about 200 μm diameter. The design of CPI module is the key to achieve the goal for accurate projection of 2D fluorescence information to 3D surgical field. In this regard, there are two critical points: (1) the design of the co-axial projection system and (2) the probe tip tracking method of the FSP system. Figure 6a illustrates the basic light path diagram of a co-axial projection system. O_1_ is the center of the optical axis of the RGB camera, whereas O_2_ is the center of the optical axis of the projector. With the projector and the camera symmetrically positioned with respect to the beam splitter, the image planes of the projector and the camera are conjugated. The pixels on the camera and on the projector establish a one-to-one correspondence so that any information acquired by the camera can be accurately projected onto the 3D object surface by the projector in situ. In Fig. 6a, for example, as long as points P, H and Q are captured by the camera, their corresponding fluorescence intensities can be orthotopically projected onto these points with accuracy. Figure 6b illustrates our specially designed probe tip tracking method. The probe is equipped with four fiducial markers (a, b, c, d). Line “a-b” and line “c-d” in 3D space intersect at the probe tip T. Line “a'-b'” and line “c'-d'” are their images on the camera 2D image plane. Therefore, T’, the intersection of “a'-b'” and “c'-d'”, is easily located on the image plane. Ray “O_1_-T'” always points to the probe tip T in 3D space, no matter how T moves along “M–N”. When co-axial projection is integrated, ray “O_1_-T'” is also the projection light ray, so that an accurate projection of the probe tip in 3D space is achieved. In conclusion, the co-axial projection system enables one-to-one mapping between any pixel on the camera and its corresponding point of projection on the 3D object, and the specially designed probe tip tracking method provides an easy and accurate approach for probe tip tracking. The combination of these two strategies ensures accurate projection from a 2D plane to a 3D space for the FSP system. The CPI comprises a visual projector (Beijing Wintech, China) which has a pixel resolution of 912 × 1140 and maximal working distance of 1000 mm, an RGB camera (Pixel: 960 × 1280, Shenzhen MindVision, China) mounted with a 12 mm focal lens (NAVITAR, US), and a 50/50% beam splitter (PHTODE, China). A rectangle frame of 50 mm × 50 mm is projected onto the patient surface to mark the region of interest. A user interface is designed with Python software (JetBrains, Czech).

### FSP system performance characterization

Considering that the fluorescence emission spectrum of PGs closely resembles that of ICG, we characterize the sensitivity of the FSP system using ICG dilute solutions at different concentrations [23, 35]. 15 mg ICG powder (Shanghai Macklin, China) is dissolved into some pure water and diluted. The concentrations of the ICG solution are: 1290 pM, 645 pM, 322 pM, 161 pM, 80 pM, 40 pM, 20 pM, 10 pM, 5 pM, 2.5 pM, 1.25 pM, 0.625 pM and 0.3125 pM. Pure water is set as control and used to measure the noise floor of the photodiode. The measurement procedure is as follows: (1) install the probe on a platform and dispense a certain amount of the ICG solution to be measured into a disposable container; (2) adjust the vertical and horizontal positions of the probe to make the excitation laser focus on the liquid surface at center of the container; (3) move the probe to acquire fluorescence intensity data at three random positions. The continuous measurement time of each point is one second, and the sampling frequency is about 50 Hz. The final data are obtained by averaging the data during this period.

The FSP system constructs a fluorescence image by correlating real-time detected autofluorescence signal with the moving probe position. Therefore, spatial resolution for fluorescence detection which decides the smallest distance when two fluorophores could be distinguished, and projection accuracy for in situ display onto the surgical site are critical parameters that will impact the image reconstruction performance of FSP system. Dynamic delay is another critical parameter since it determines whether FSP could provide real-time navigation intraoperatively.

**Fig. 7:**
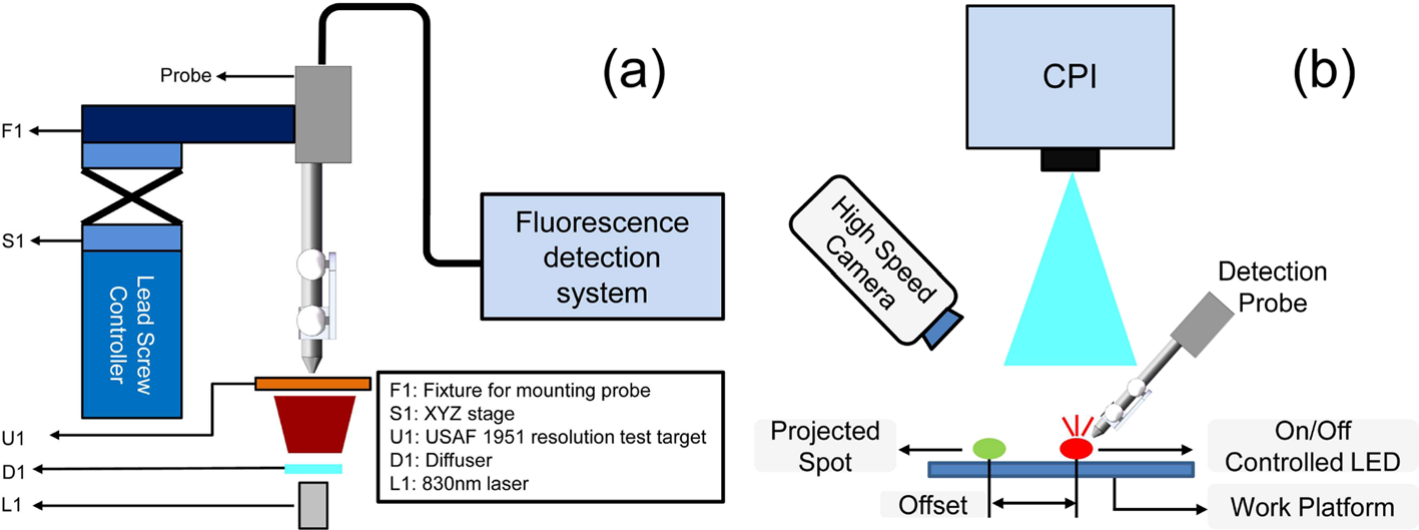
Schematic of spatial resolution for detection and dynamic performance characterization system. **a** Schematic of spatial resolution measurement system. **b** Schematic of FSP dynamic performance characterization system

Spatial resolution for fluorescence detection is measured using a phantom that comprised an 830-nm laser (OCEANHOOD, China), a diffuser (Thorlabs, US) and a negative USAF resolution target (Thorlabs, US) [36] (Fig. 7a). The diffused light from the laser transmits through the resolution target and is detected by the probe, which is mounted on a motor driven platform. The platform is set to scan over a certain group of elements of the resolution target at a constant speed from low-resolution group to high-resolution group. At the same time, the system continuously records the fluorescence intensity at each point during the scanning. The data acquisition speed is set to ensure that the Nyquist sampling law would be met at the highest resolution group which indicates higher signal frequency.

An measuring area of 50 mm × 50 mm and working distance ranging from 35 to 55 cm are considered adequate for PG detection [25]. Therefore, projection accuracy and spatial resolution for imaging are characterized under such conditions. A rectangle is projected onto the working platform covered by a coordinate graph paper to mark the region of interest. The two orthogonal directions of the coordinate graph paper are defined as the *x-* and *y*-axis. System is characterized under five different working distances: 35 cm, 40 cm, 45 cm, 50 cm and 55 cm. The probe is mounted on a fixture at a fixed angle. The tip of the probe is placed at the center of crosses at the interval of 10 mm and held for seconds. The RGB camera detects the fiducial markers and the four ball centers are calculated through circle fitting algorithm. The intersection of the two lines determined by the ball centers is calculated to determine the (*x, y*) pixel coordinate of the probe. Then CPI projects a green solid circle on paper and the center of this circle indicates the probe position. A visible image of the projecting circle is photographed and the gravity center coordinate is calculated as the actual projecting point by software. Coordinates bias between the projecting point and each cross center are calculated along the two orthogonal axes and they are notated as *x* coordinate error and *y* coordinate error, respectively. The projection distance error is calculated using the Euclidean metric under a Cartesian coordinate system. A negative sign is assigned to the distance error when the projecting point belongs to the lower half plane while the projecting plane is divided along the *x*-axis or belongs to the left half plane while it is divided along the *y*-axis. The distance error histogram is fitted with Gaussian distribution, and the spatial resolution for imaging is assessed by the Sparrow criterion, which defines the smallest target separation discernible through amplitude modulation [30].

The dynamic performance is determined by measuring the time between the start of the fluorescence detection and the successful projection of the results (Fig. 7b). An electrically controlled red LED is detected by the probe, and the intensity of the detected light is projected on the work platform as a spot 5 cm away from the detected probe tip position in order for easier distinguishing. A high-speed camera records the whole process and the time delay is calculated by analyzing the video frames in MATLAB software. The measurement is repeated 15 times and data are averaged as the final time delay.

### Surgical simulation on a PG-simulating phantom

Ambient room lights have little impact on the FSP detection sensitivity and are allowed to be kept on during PG detection. However, surgical shadowless lights should be off or moved aside because some of these lights might have wide range wavelength overlaps with PG autofluorescence. PG autofluorescence and ICG solution fluorescence intensity has been compared and it is suggested that 0.02 μg/ml (about 25 pM) ICG can be used as PG-simulating phantom to evaluate the capability for PG detection [23].

During a thyroidectomy, the PGs might be in two different states after thyroid is exposed. In one situation, the PGs are totally exposed, while in the other situation, they may be covered by adipose or connected tissue. We simulate the PGs of different states in our studies. A 6-mm-diameter flat-bottomed glass cup filled with 25 pM ICG solution is used to simulate the fully exposed PG phantom. In order to simulate PG phantom covered by adipose of different thicknesses, we design a fixture to install the container filled with the ICG solution at 10-degree tilt angle from the horizontal plane. RTV silicone (Hong Ye Silicone, Shenzhen) mixed with appropriate India ink as absorber and TiO_2_ as scattering materials is poured and solidified in vacuum over PG phantom to simulate adipose as shown in Fig. 3b. Another two containers that dispensed with pure water are used as negative control.

The surgical scenario of fluorescence-guided PG localization is simulated in the laboratory: a ring white light source is installed to simulate interference from operating room ambient lights. The PG phantom is installed on the neck of a human model, and an experienced user uses the probe to scan on the phantom. During the measurement, a pseudo-color image constructed from the normalized fluorescence intensity is projected onto the phantom surface. Ten times measurements of the phantom are repeated and the projection images are record by an RGB camera fixed in position. The area marked as black circle in Fig. 3d is indicated as PG by FSP and its position is compared with the red circle fitted based on the fluorescence image obtained by LSFI. The images are processed using software ImageJ (Fiji, US).

### Ex vivo* tissue validation*

The excised tissues are supplied from the First Affiliated Hospital of University of Science and Technology of China after the approval from the IRB (Hefei First Affiliated Hospital, 2021KY NO. 135). The PG, lymph node and thyroid gland tissues are from a 57 years old female patient who had renal induced secondary hyperparathyroidism and accepted parathyroidectomy.

The operating steps are as follows: (1) scan on the lymph node and thyroid tissue to get the background signal, which is determined by the maximal value at all tested positions; (2) start to scan at any target tissue using the probe. The real-time detected fluorescence intensity is normalized to background and mapped over the tissue to form a pseudo-color image; (3) green visible light would be projected onto the detected point where the detected value exceeds the cut-off value for PG which is set to be 1.5 [25]. A projection overlay image generating process comprises three consecutive tasks coordinated by a software package at a framerate of 30 measurements/second: data acquisition, processing, and projection. As the FSP probe scans over the surgical field, the acquired fluorescence intensity level at each location of the target tissue is converted into a visible color and projected back to the actual location for an extended duration so that the fluorescence information remains visible even after the probe moves away. Pseudo-color image and projected green visible image can be alternatively displayed to reduce interference with each other by manual switching on the user interface.

### Statistics analysis

The OriginPro software (9.7, OriginLab, USA) is used for statistical analysis. The final measurement results are expressed by mean ± standard deviation (SD).

## Data Availability

The datasets used and/or analyzed during the current study are available from the corresponding author on reasonable request.

## Abbreviations

NIR: Near-infrared
PGs: Parathyroid glands
FSP: Fluorescence scanning and projection
ICG: Indocyanine green
SNR: Signal-to-noise ratio
IoU: Intersection over unit
OTIS: Overlay tissue imaging system
FOV: Field of view
CPI: Coaxial projective imaging
FWHM: Full width at half-maximum
LSFI: Laser scanning fluorescence imaging
RTV: Room-temperature vulcanizing
OD: Optical density
APD: Avalanche photodiode
LD: Laser diode
DMD: Digital micromirror device
CMOS: Complementary metal oxide semiconductor
